# Jejunum-derived NF-κB reporter organoids as 3D models for the study of TNF-alpha-induced inflammation

**DOI:** 10.1038/s41598-022-18556-3

**Published:** 2022-08-24

**Authors:** Hellen Daghero, Flora Doffe, Belén Varela, Victoria Yozzi, José Manuel Verdes, Martina Crispo, Mariela Bollati-Fogolín, Romina Pagotto

**Affiliations:** 1 Cell Biology Unit, Institut Pasteur de Montevideo, Mataojo 2020, 11400 Montevideo, Uruguay; 2 INSERM, UMR 1186, Integrative Tumor Immunology and Immunotherapy, Gustave Roussy, Faculty of Medecine, University Paris-Saclay, 94805 Villejuif, France; 3 Pathology Unit, Department of Pathobiology, Faculty of Veterinary, Universidad de la Republica, Route 8, Km. 18 and Route 102, CP 13000 Montevideo, Uruguay; 4 Laboratory Animal Biotechnology Unit, Institut Pasteur de Montevideo, Mataojo 2020, 11400 Montevideo, Uruguay

**Keywords:** Biological techniques, Biotechnology, Cell biology, Drug discovery, Microbiology

## Abstract

Inflammation is an important process for epithelial barrier protection but when uncontrolled, it can also lead to tissue damage. The nuclear factor-kappa light chain enhancer of activated B cells (NF-κB) signaling pathway is particularly relevant in the intestine, as it seems to play a dual role. Whereas NF-κB protects intestinal epithelium against various noxious stimuli, the same pathway mediates intestinal inflammatory diseases by inducing pro-inflammatory gene expression. The availability of appropriate in vitro models of the intestinal epithelium is crucial for further understanding the contribution of NF-κB in physiological and pathological processes and advancing in the development of drugs and therapies against gut diseases. Here we established, characterized, and validated three-dimensional cultures of intestinal organoids obtained from biopsies of NF-κB-RE-Luc mice. The NF-κB-RE-Luc intestinal organoids derived from different intestine regions recreated the cellular composition of the tissue and showed a reporter responsiveness similar to the in vivo murine model. When stimulated with TNF-α, jejunum-derived NF-κB-RE-Luc-reporter organoids, provided a useful model to evaluate the anti-inflammatory effects of natural and synthetic compounds. These reporter organoids are valuable tools to explore the epithelial TNF-α-induced NF-κB contribution in the small intestine, being a reliable alternative method while helping to reduce the use of laboratory animals for experimentation.

## Introduction

The intestinal epithelium is a highly specialized tissue exerting major functions in the organism. It participates in the absorption of nutrients, water, and electrolytes, acts as a barrier preventing the entry of harmful substances into the body, and interacts with the microbiota, contributing to the defense against pathogens and modulating the immune response^1,2^. This simple epithelium comprises different specialized intestinal epithelial cells (IECs), including enterocytes, Goblet cells, enteroendocrine cells, Paneth cells, M cells, and Tuft cells, all of which differentiate from common epithelial stem cells^3^. The IECs are functionally different and essential for maintaining intestinal homeostasis by separating the intestinal lumen from the underlying lamina propria and by controlling the crosstalk between luminal microbiota and subjacent immune cells^4,5^.

Nuclear factor-kappa light chain enhancer of activated B cells (NF-κB) refers to a family of evolutionarily conserved transcription factors that regulate a wide range of biological processes, including cell growth and survival to immunity and inflammation^6^. Activation of NF-κB depends on phosphorylation-induced ubiquitination of IκB proteins. Once the IκB inhibitor is degraded, NF-κB translocates to the nucleus, where it binds to κB-cis-acting elements present on a subset of genes and triggers transcriptional activity^7^. The NF-κB signaling pathway is involved in various intestinal biological processes such as cellular proliferation, differentiation and survival, inflammation, and carcinogenesis^8,9^. This transcriptional system seems to play a dual role at the intestinal epithelium by exerting deleterious effects as well as protective functions, depending on the stimulus received^10,11^. For instance, numerous studies have shown that a sustained NF-κB activity in the intestine acts as a pro-inflammatory factor and participates in the development of chronic inflammatory diseases, like Inflammatory Bowel Disease^12^. On the other hand, diverse genetic approaches have demonstrated that NF-κB activation on intestinal epithelial cells might be necessary to maintain epithelial integrity and homeostasis in the gut^13^.

In vitro models of the intestinal epithelium have proved to be valuable tools to study the contribution of NF-κB signaling in different contexts. In particular, our group has generated and characterized human colon adenocarcinoma (HT-29 and Caco-2) reporter cell lines for functional studies of NF-κB activation. These reporter cell lines were successfully used for the screening of probiotic bacteria and antitumor peptides with anti-inflammatory effects^14^. However, the use of traditional cell line cultures has the drawback of being genetically modified, altering their physiological properties and do not adequately resemble the natural three-dimensional (3D) environment of cells, or recreate the tissue´s original cellular diversity, sometimes providing misleading and non-predictive data as in vivo responses^15^.

Recent advances in stem cell biology have enabled long-term culturing of organotypic intestinal tissues derived from tissue-resident or pluripotent stem cells^16,17^. These three-dimensional and self-organizing structures, known as intestinal organoids, recapitulate the architecture, functionality, and genetic signature of the tissue^18^. They present substantial advances over traditional 2D cultures: they have unlimited natural replicative capacity; they recreate the cellular diversity of the tissue; they mimic the in vivo state more closely than 2D cultures and have higher predictive value^19,20^.

The present work aimed to establish and characterize intestinal reporter organoids for NF-κB activity. Intestinal organoids were derived from isolated intestinal crypts of the NF-κB-RE-Luc transgenic mice. This transgenic model generated by Carlsen et al.^21^ has a randomly integrated DNA construct consisting of three NF-κB binding sites from the immunoglobulin κ light chain promoter driving the firefly luciferase expression. These new reporter organoids were validated using compounds known to inhibit different NF-κB signaling steps, providing a useful ex vivo model to evaluate the NF-κB activity in intestinal epithelial cells.

## Results

### Size, morphology and replicative capacity differ between organoids derived from small intestine and colon

Intestinal organoids were established from isolated crypts obtained from the small and large intestine regions of male and female NF-κB-RE-luc transgenic mice. The same procedure and the biopsy size (3 cm pieces of tissue) were used for obtaining duodenum, jejunum, ileum, and colon-derived crypts. Nevertheless, the efficiency of the isolating procedure varied between the intestinal regions, being the ileum the segment with the lowest number of crypts recovered (data not shown).

**Figure 1:**
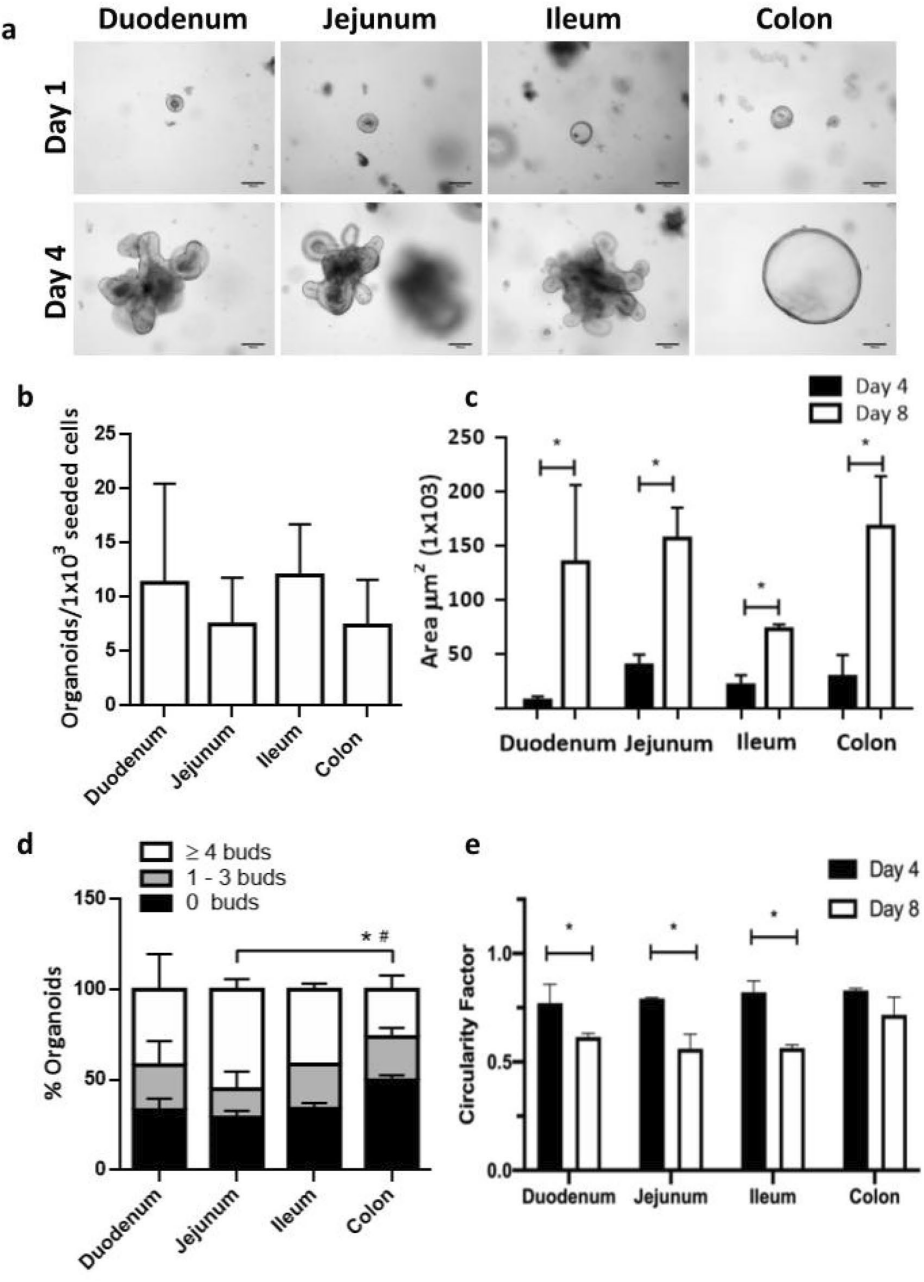
Culture of intestinal reporter organoids for NF-κB activity. Intestinal organoids were obtained from crypts isolated from duodenum, jejunum, ileum and colon of the NF-κB reporter mice as described in “Methods” section. (**a**) Representative bright-field images of organoids at day 1 and day 4 of culture. (**b**) Replicative capacity of organoids expressed as spheroids generated from 1000 seeded single epithelial cells was quantified on day 4. (**c**) Growth was evaluated by measuring the area of the organoids at days 4 and 8 of culture; *P < 0.05, significant differences between the same intestinal region-derived organoids at day 4 and 8 of culture. (**d**) The number of buds per organoid was manually estimated at day 8 of culture and the percentage of organoids without buds, with 1–3 buds or with more than 4 buds was compared between each type of organoid; *P < 0.05, significant differences in the percentage of organoids with more than 4 buds; #*P* < 0.05, significant differences in the percentage of orgnaoids without buds. (**e**) Circularity factor was measured in organoids at days 4 and 8 of culture. A value of 1.0 indicates a perfect circle. *P < 0.05, significant differences with respect to the same intestinal region-derived organoids at day 4. Two biological replicates (organoids from two different animals) for each intestinal region were analysed. Each biological replicate was done in triplicate.

After 24 h of culture, crypts closed themselves, leading to a cyst or a spherical cellular structure (spheroid) (Fig. 1a, Day 1). By day 4 of culture, spheroids grew in size and complexity, leading to the characteristic 3D architecture of cyst with a central lumen flanked by a simple polarized epithelium and the basal side of the cells oriented toward the outside. Organoids derived from the small intestine showed multiple new crypt-like structures or buds emerging from the center to the surrounding matrix, whereas organoids derived from the colon showed a symmetric spherical shape (Fig. 1a, Day 4).

To better characterize the different types of organoids, morphological and growth parameters were analyzed from single-cell dissociated organoid cultures (Fig. 1b–e). The number of organoids generated from 1000 single epithelial cells seeded, was evaluated after 4 days in culture. This parameter ranged between 11.33 ± 9.15 to 7.35 ± 4.20 organoids, with no statistically differences among the different intestinal organoids (P = NS) (Fig. 1b). Regarding growth parameters, organoids developed from single cells took more days to grow in size, compared with the culture isolated from crypts. By day 8, all organoids had increased their area several times compared with day 4 (Fig. 1c) and their aspect resembled those of organoids derived from intestinal crypts at day 4 of culture (data not shown). There was a significant difference between jejunum and colon-derived organoids with respect to the numbers of buds developed in each organoid. Jejunum-derived cultures presented a higher percentage of organoids with more than four buds (55.02 ± 5.76%, for jejunum; 26.25 ± 7.86% for colon, *P < 0.05), whereas colon-derived organoids showed a higher percentage of structures without buds (29.12 ± 3.82% for jejunum; 49.73 ± 2.82% for colon, #*P* < 0.05) (Fig. 1d). Circularity was in accordance with these results; small intestinal derived-organoids significantly decreased their circularity at day 8 of culture compared with day 4 (*P* < 0.05), whereas colon-derived organoids maintained the highest value for this parameter (day 8, 0.72 ± 0.19 circularity factor) throughout the evaluated period (Fig. 1e).

**Figure 2:**
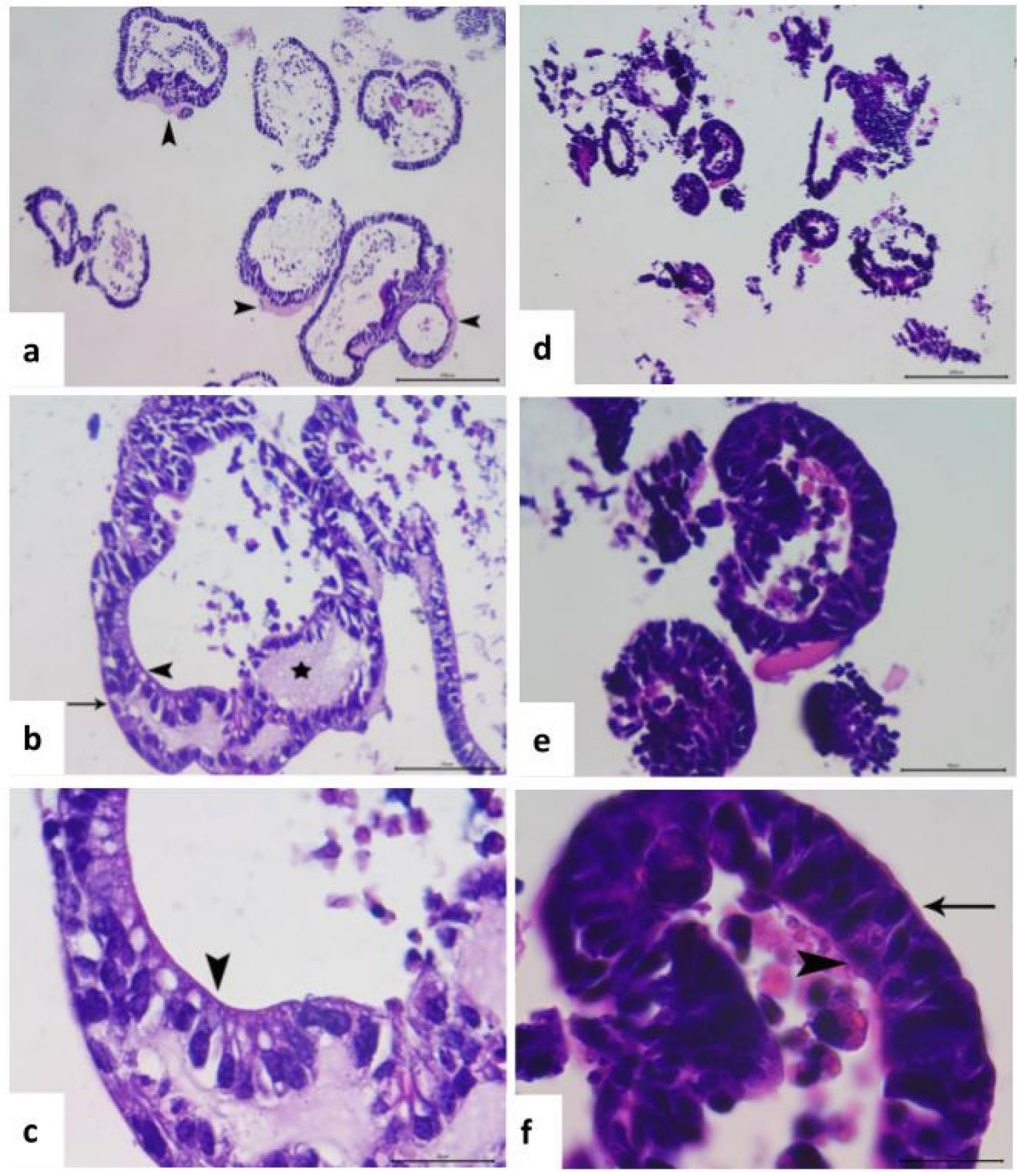
Histological analysis of NF-κB reporter intestinal organoids. Colon-derived (**a**–**c**) and jejunum-derived (**d**–**f**) organoids. (**a**) Cluster of colonoids of several sizes. Multiple epithelial ductal structures (columnar epithelium), associated by amorphous eosinophilic matrix (arrowhead). Hematoxylin–eosin staining (HE), 10×. Bar = 200 µm. (**b**) Colonoid epithelial duct showing different cell types described. Columnar epithelium organized from the subtle eosinophilic extracellular matrix (arrow), with amorphous and diffusely vacuolated eosinophilic material (asterisk), presence of some goblet-like cells in the luminal ductal surface were typical (arrowhead). HE, 40×. Bar = 50 µm. (**c**) High magnification of (**b**), showing epithelial cells (typical rounded and intense basophilic nuclei), interspersed with goblet-like cells with vacuoles in the luminal ductal surface (arrowhead). HE, 100×. Bar = 20 µm. (**d**) Cluster of jejunum-derived organoids of several sizes. Multiple epithelial ductal structures (columnar epithelium).HE, 10×. Bar = 200 µm. (**e**) Epithelial ducts showing similar cell types described in (**b**), with detachment of necroapoptotic cells in the ductal lumen. HE, 40×. Bar = 50 µm. (**f**) High magnification of (**b**), showing epithelial cells organized from the basal extracellular matrix (arrow), with less abundant goblet-like cells (arrowhead). HE, 100×. Bar = 20 µm.

Histological analysis of organoids derived from jejunum and colon revealed multiple well-differentiated and preserved ductal structures, organized from the outside by a delicate basement membrane (Fig. 2). In colonoids a remnants of a strongly eosinophilic, amorphous matrix with a diffusely vacuolated appearance is observed. In both organoids the epithelial ducts have at least one-cell continuous layer of columnar-type epithelial cells, supported at their basal pole by a subtle eosinophilic extracellular matrix, with round to ovoid strongly basophilic nuclei. In those cases where clusters of ductal structures were observed, the epithelium were constituted by a epithelial cell multilayer (from 2 to 5 cells thickness), with similar histologic appearance to the previous description, always maintaining the ductal organization. Frequently, on the luminal border of these ducts, other less abundant rounded cells were observed, interspersed among the epithelial cell layers. These cells were identified by their clear eosinophilic foamy cytoplasm, with the presence of larger vacuoles, reminiscent of goblet cells. In the lumen of the ducts, it was also frequent to observe detachments of necro-apoptotic cellular masses, being rounded in appearance, with cariolisis, and with abundant and foamy cytoplasm (ghost cells).

**Figure 3:**
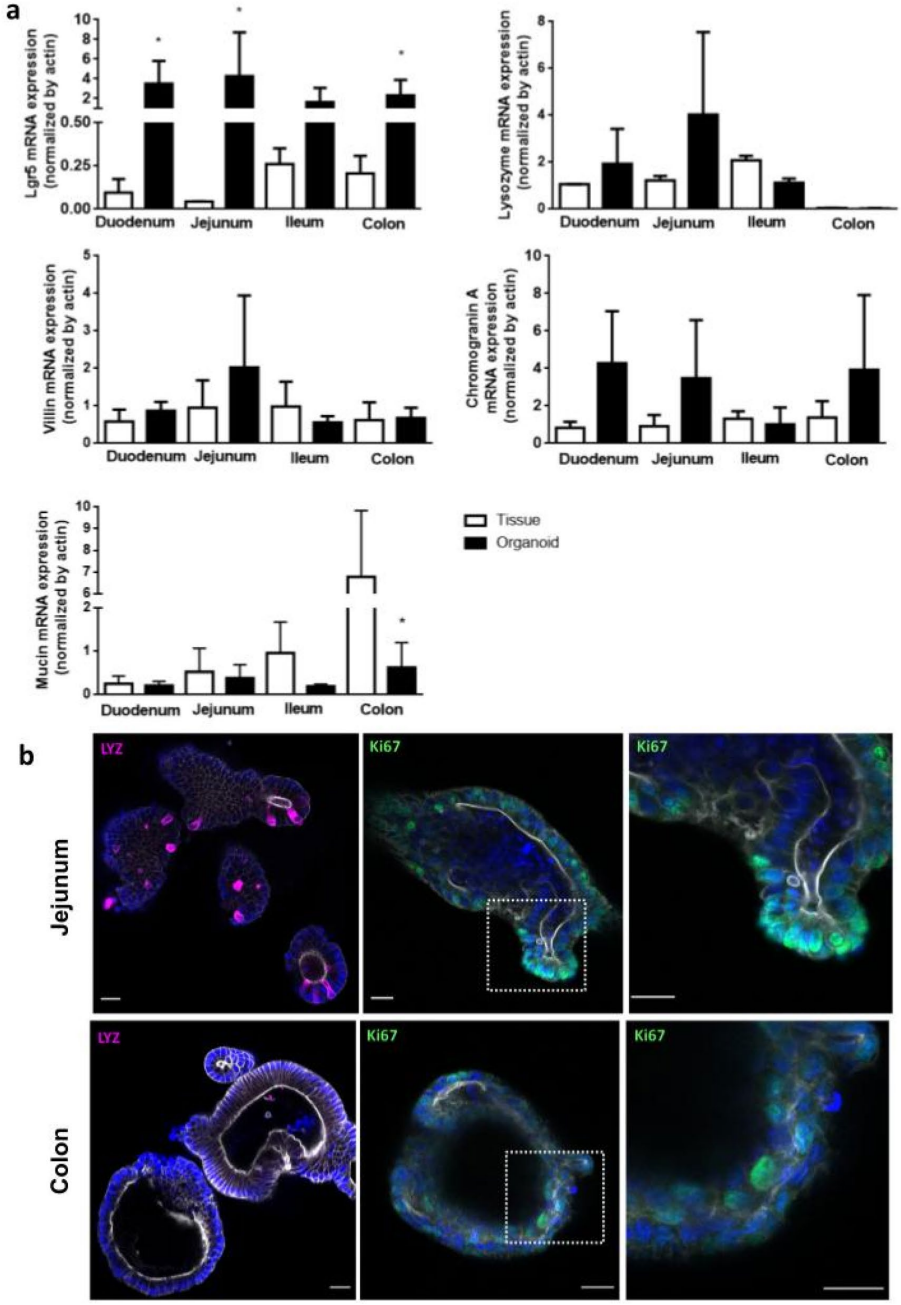
Specific intestinal markers for cell populations in organoid cultures. (**a**) The presence of the major intestinal cell types where analyzed by qPCR for specific intestinal markers: intestinal stem cells (Lgr5), enterocytes (Villin); Paneth cells (Lysozyme), Goblet cells (Mucin 2) and enteroendocrine cells (Chromogranin A). The mRNA levels of each marker were normalized by actin expression. Data was expressed as mean ± SD of two (tissue) or at least three (organoids) different animals. *P < 0.05, significant differences between the intestine tissue and the corresponding derived-organoid. (**b**) Detection of Paneth cells (Lysozyme positive cells in magenta) and proliferating cells (Ki67 positive cells in green) in organoids derived from small intestine and colon by immunofluorescence. Nuclei in blue; actin in gray. Scale bar = 20 µm.

Characterization of the epithelial cell population was performed by quantitative analysis of mRNA expression from organoids and intestinal tissue samples. Five specific genes, representative for the main intestinal epithelial cell types were evaluated, Lgr5 (intestinal stem cell), Lysozyme (Paneth cell), Villin (enterocytes), Chromogranin A (enteroendocrine cells), and Mucin 2 (Goblet cells). As shown in Fig. 3a, the expression pattern of Villin, Chromogranin A and Lysozyme were similar between tissue and organoids from the same intestinal region. Lysozyme could not be detected in samples from tissues and organoids derived from the colon, since Paneth cells do not populate this intestinal region. Mucin 2 expression was reduced in colon-derived organoids, compared with the same tissue (*P* < 0.05). Of note, except for ileum, Lgr5 levels were higher in organoids compared with the corresponding tissue (*P* < 0.05). The presence of Paneth cells in small intestine-derived organoids were corroborated at the protein level by immunodetection of Lysozyme, whereas actively proliferating cells were found in both organoids, as indicated by the presence of Ki67 positive cells (Fig. 3b).

### NF-κB reporter ileum- and jejunum-derived organoids are most sensitive to TNF-α stimulus

**Figure 4:**
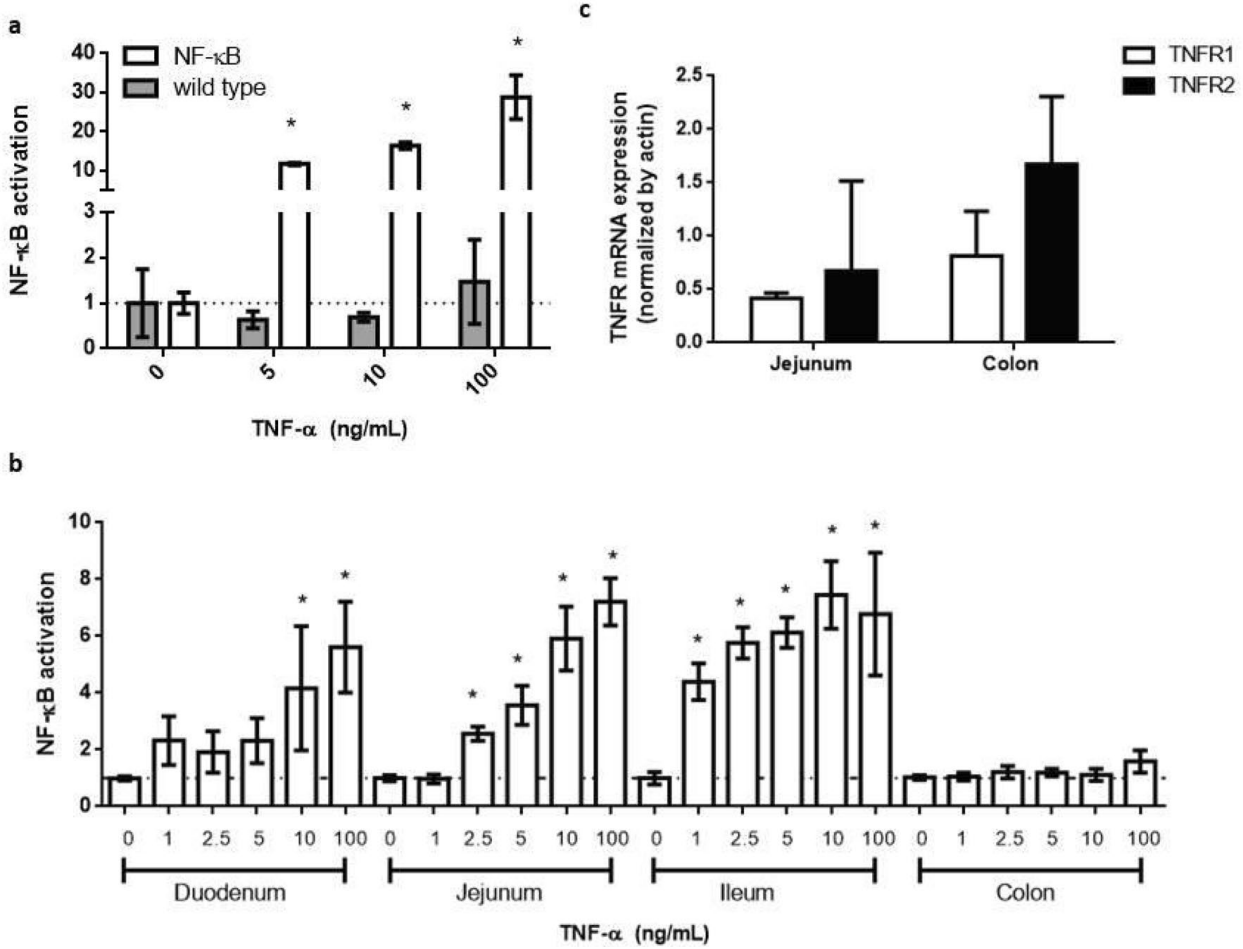
Response of NF-κB reporter intestinal organoids to TNF-α. Organoids were stimulated with different concentrations of TNF-α. Luciferase activity was evaluated after 24 h of incubation, and NF-κB activation was expressed as fold change with respect to the unstimulated control. (**a**) Reporter activity from small intestine-derived organoids was specific for organoids obtained from NF-κB reporter mice (white), as wild type organoids (gray) did not show luciferase activity; (**b**) NF-κB reporter responsiveness varied among the different intestine region-derived organoids. The jejunum-derived organoids showed the higher response, whereas colon organoids were unresponsive. Results from one representative experiment are shown in a and b, and data was expressed as mean ± SD of triplicates; (**c**) both colon- and jejunum-derived organoids expressed mRNA for TNF receptors. The mRNA expression levels of each receptor subtype were normalized by actin expression and data was expressed as mean ± SD of at least three organoids obtained from different animals. *P < 0.05, significant differences with respect to the unstimulated control.

The activation of the NF-κB transcription factor was evaluated by stimulating transgenic and wild-type organoids derived from the small intestine with different concentrations of Tumor Necrosis Factor-alpha (TNF-α) as the pro-inflammatory stimulus. After 24 h of incubation, luciferase activity was measured. As shown in Fig. 4a, luciferase activity was only detected in NF-κB-RE-Luc organoids, which showed a concentration-dependent response to the inflammatory stimulus. The maximum NF-κB activation levels were attained at 100 ng/mL TNF-α, with more than 20 fold change increase compared with the control value (un-stimulated organoids) (*P* < 0.05), confirming the ability of the organoids to report the activation of NF-κB signaling pathway.

To better characterize the model, the responsiveness of organoids derived from duodenum, jejunum, ileum and colon were also evaluated with TNF-α. The sensitivity of the reporter response varied between the intestinal regions and within the same region, among different cultures (see supplementary material, Table S1 and Figs. S1 and S2). Ileum- and jejunum-derived organoids were the most sensitive to the stimulus, showing a significant increase in the reporter signal with concentrations equal or higher than 1 and 2.5 ng/mL of TNF-α, respectively. On the opposite side, it was not possible to detect any reporter signal from colonic organoids. Organoids obtained from duodenum produced a detectable luciferase reporter response only at higher concentrations of TNF-α (at 10 and 100 ng/mL) (Fig. 4b).

Organoids obtained from a responsive (jejunum) and unresponsive (colon) intestinal region were further studied for the presence of TNF-α Receptor 1 and TNF-α Receptor 2 (TNF-R1 and TNF-R2). Both intestinal organoids were able to express mRNA from TNF-R1 and TNF-R2 (Fig. 4c), which suggests that they could bind the TNF-α and respond to this stimulus.

Regarding the stability of the reporter system over time, the NF-κB reporter activity of jejunum-derived organoids was evaluated with different concentrations of TNF-α at different passages. As indicated in Table 1, the concentration of TNF-α that gives half-maximal response (EC50 values) were similar among all the passages, indicating a stable reporter activity at least during 16 passages. Beyond this fact, we defined passage 12 as the maximum passage number for other assays.

**Table 1:** Stability of NF-κB reporter activity from jejunum-derived organoids.

Parameter	Jejunum-derived organoids			
	Passage 9	Passage 12	Passage 13	Passage 16
EC50	4.86	3.81	3.77	3.16
CI 95%	2.42–9.75	0.98–14.9	1.37–10.35	0.78–12.76

Organoids were stimulated with TNF-α during 24 h at different passage numbers. The NF-κB activation was measured by detection of luciferase activity. EC50 and its 95% confidence interval (ng/mL) were calculated using non-linear regression 3-parameter fit with GraphPad Prism.

**Figure 5:**
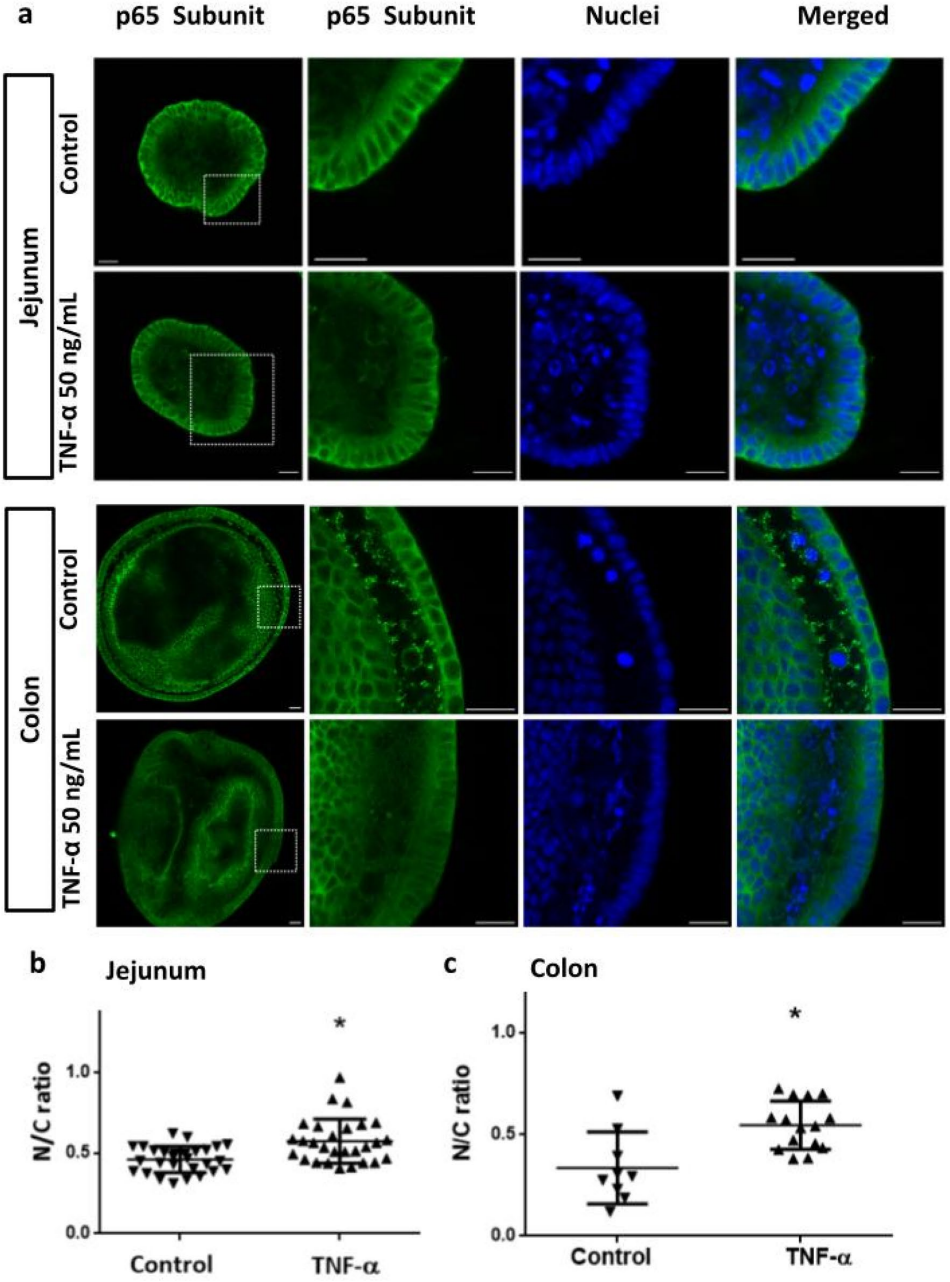
TNF-α induces NF-κB translocation into the nucleus of intestinal organoids. Organoids derived from jejunum and colon were stimulated with TNF-α (50 ng/mL) during 3 h. Unstimulated organoids were used as control. NF-κB was detected by using an anti-p65 antibody (green). Nuclei were stained with methyl green (blue). In both stimulated organoids, the NF-κB signal was more homogeneously distributed between nucleus and cytoplasm compared to the controls, where non-labeled nuclei predominate (**a**). Nuclear translocation of NF-κB was quantified using the green signal intensity ratio between the nuclei and the cytoplasm (N/C ratio) (**b**, **c**). Individual values in the graph represent one organoid measurement. Scale bar = 20 µm. Data was expressed as mean ± SD. *P < 0.05, significant differences with respect to the unstimulated control.

Direct measurement of NF-κB pathway functionality was evaluated by comparing TNF-α-induced translocation of NF-κB in jejunum- and colon-derived organoids. Organoids were stimulated with 50 ng/mL of TNF-α for 3 h and the nuclear translocation of NF-κB p65 subunit was analyzed by confocal microscopy (Fig. 5). Images show that in TNF-α-stimulated organoids, NF-κB p65 signal is more homogeneously distributed between the nucleus and the cytoplasm, while in unstimulated controls, p65 signal predominate in the cytoplasm, being more evident the presence of non labeled-nuclei (Fig. 5a, jejunum and colon). These findings suggest that TNF-α induced the translocation of the p65 subunit to the nucleus, while in non-stimulated cells, NF-κB was mainly detected in the cytoplasm. Nuclear translocation was quantified by calculating the ratio between the signal intensity of the nucleus and the cytoplasm (N/C ratio). In both jejunum and colon-derived organoids, the N/C ratio was increased (*P* < 0.05) in the TNF-α-stimulated organoids compared to non-stimulated conditions, indicating nuclear translocation of NF-κB transcription factor, a key step in the performance of the reporter assay (Fig. 5b,c).

These results confirmed the presence of a functional NF-κB signaling pathway in both jejunum- and colon-derived organoids. Thus the lack of reporter activity in colon-derived organoids would not be associated with an alteration of this signaling pathway.

**Figure 6:**
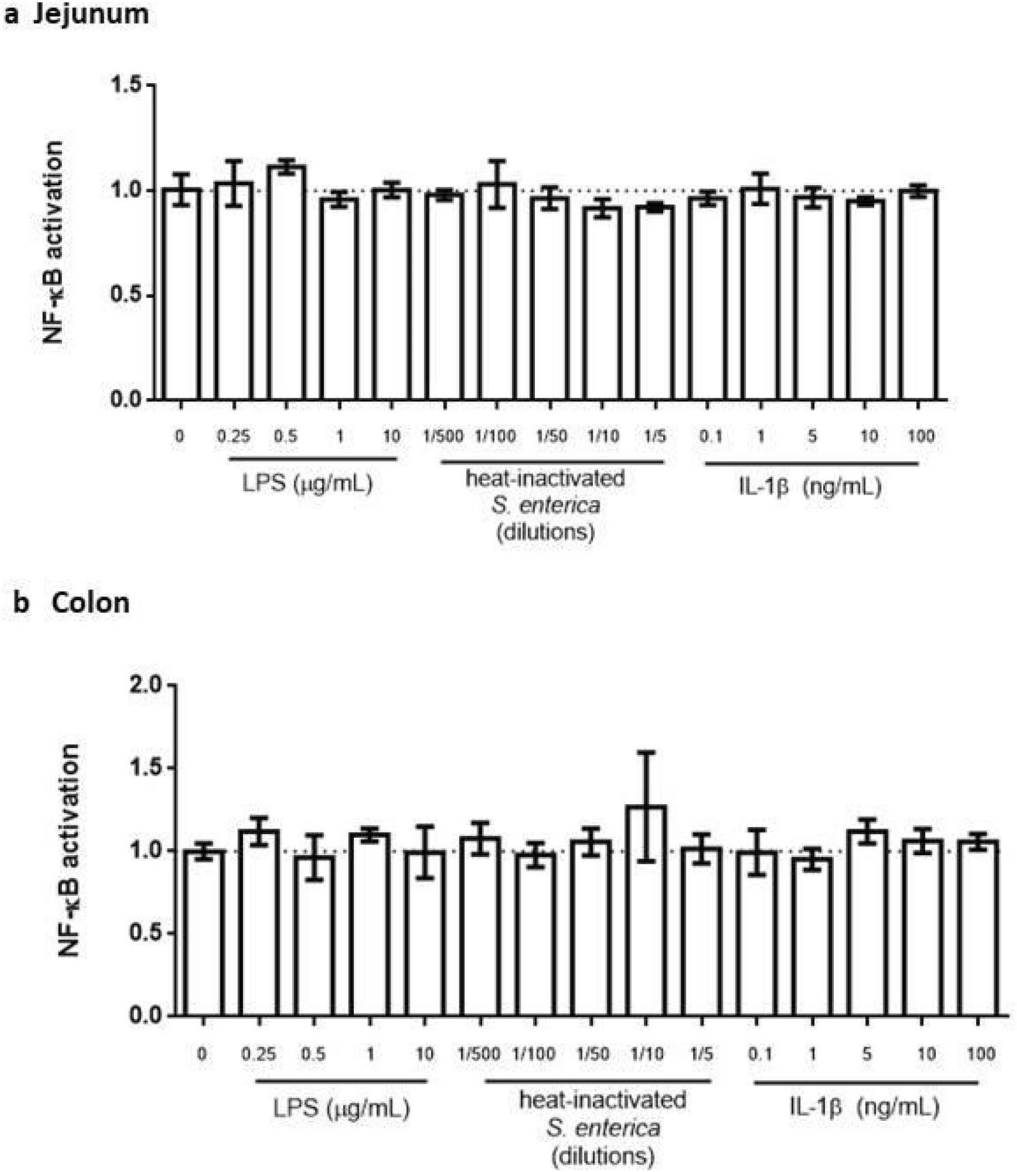
Activation of NF-κB with different proinflammatory stimuli. NF-κB reporter organoids derived from jejunum (**a**) and colon (**b**) were stimulated during 24 h with LPS, IL-1β and heat-inactivated *Salmonella enterica*. NF-κB activation was determined as previously described. There were no significant differences between the stimulated conditions and the unstimulated group. Data was expressed as mean ± SD of triplicates from one representative experiment.

In addition to TNF-α, the intestinal epithelium is frequently exposed to other inflammatory stimuli. We next evaluated the ability of organoids to respond to three well known intestinal inflammatory compounds: Lipopolysaccharide (LPS), a heat-inactivated *Salmonella enterica* extract^22^ and interleukin 1 beta (IL-1β). Neither of them induced a detectable reporter response in the NF-κB-RE-Luc organoids, regardless of the intestinal region analyzed (Fig. 6).

### Natural and synthetic compounds modulate TNF-α- induced NF-κB activation in jejunum-derived organoids

Based on our results, TNF-α induced a potent luciferase response at 10 ng/mL in reporter organoids derived from jejunum. Therefore to validate the model, we selected this system and tested synthetic and natural compounds known to interfere with the NF-κB signaling pathway.

**Figure 7:**
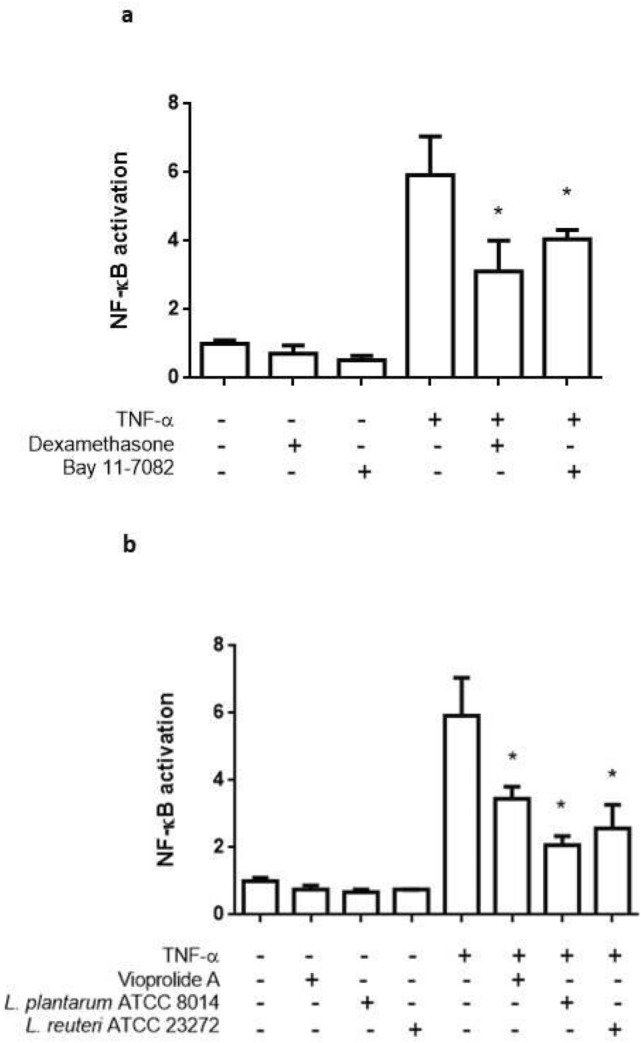
Validation of the NF-κB-RE-Luc organoids with anti-inflammatory compounds. NF-κB reporter organoids were stimulated with TNF-α 10 ng/mL and incubated with synthetic and natural antiinflammatory compounds for 24 h. NF-κB activation was determined as previously described. Cells without treatment and cells treated only with TNF-α or the different compounds were included as controls. (**a**) Known synthetic inhibitors of NF-κB pathway Dexamethasone and Bay 11-0782; (**b**) conditioned medium from probiotic *L. plantarum* and *L. reuteri* and the compound Vioprolide A were used as natural anti-inflammatory compounds. Data was expressed as mean ± SD of triplicates from one representative experiment *P < 0.05, significant differences with respect to unstimulatedorganoids for compounds alone, or to TNF-α 10 ng/mL for compounds co-incubated with proinflammatory stimuli.

Dexamethasone (Dex) and Bay11-7082 (Bay) significantly reduced TNF-α-induced NF-κB activation, as indicated by the decrease in the luciferase activity (NF-κB activation as fold change with respect to the unstimulated control: TNF-α = 5.92 ± 1.12; Dex = 3.11 ± 0.89; Bay = 4.05 ± 0.27) (Fig. 7a). The natural peptide Vioprolide A (VioA) as well as *Lactobacillus plantarum and L. reuteri* conditioned media (CM), all were able to significantly reduce the TNF-α-induced NF-κB activation as indicated by the reduction in the reporter response, without altering the basal value, when compared with the unstimulated control (NF-κB activation in fold change respect to the unstimulated control: *L. reuteri* CM = 2.57 ± 0.71; *L. plantarum* CM = 2.07 ± 0.27; VioA = 3.45 ± 0.36) (Fig. 7b).

## Discussion

This study proposes novel NF-κB reporter intestinal organoids as a 3D model to study small intestine inflammation in health and disease.

Intestinal organoids derived from jejunum, duodenum, ileum and colon of NF-κB-RE-Luc reporter mice were successfully established from isolated intestinal crypts. Their growing capacity, evaluated through a colony forming assay, were similar regardless of the intestinal origin. On the other hand, significant morphological differences were detected between jejunum- and colon-derived organoids. A higher percentage of organoids from the jejunum presented multiple buds, whereas the colon-derived organoids were more spherical in shape, as indicated by the higher circularity factor and the higher percentage of structures without buds at day 8 of culture. It has been proposed that these morphological differences would be related to the Wnt gradient present in the medium. In the small intestinal organoids, Paneth cells would induce a sharp gradient of Wnt making the crypts to protrude from the surface whereas in colonic organoids the absence of this cell type would produce a more homogenous stimulus, favoring the spherical shape^23^. Histological analysis of jejunum- and colon-derived organoids showed cellular structures composed of a continuous layer of columnar-type epithelial cells and the presence of scattered goblet-like cells, resembling the intestinal tissue.

Regarding intestinal cell populations, organoids recreated the epithelial cellular diversity of each intestinal region, as indicated by the expression of different specific intestinal cell type markers. Nevertheless, some differences were found between organoids and tissues. The higher expression of the stem cell marker Lgr5 in most of the organoids, or the lower expression of Goblet cell marker Mucin2 in colon-derived organoids, compared to the corresponding tissue, could be a consequence of the culture conditions as Wnt and R-spondin (both factors present in the medium) favor the proliferation of intestinal stem cells over the differentiation to specialized cells^24^. The NF-κB reporter activity differed between the intestinal organoids analyzed. Whereas jejunum- and ileum-derived organoids showed a concentration–response curve to TNF-α, organoids derived from colon did not produce any detectable signal. It is known that the sensitivity of a reporter gene system may depend on multiple factors, like the site of insertion of the transgene in the DNA, the number of copies inserted, the intrinsic characteristics of the reporter protein, and the appropriate cellular machinery that allows the system's activation. TNF-α is a key player in inflammatory processes, and it is a well-known stimulus for the activation of NF-κB pathway in the intestinal epithelium^25^. It binds to and stimulates two structurally related cell surface receptors TNF-R1 and TNF-R2, both of which were expressed in the reporter organoids. Moreover, the p65 subunit of NF-κB was able to translocate to the nucleus of jejunum- and colon-derived organoids upon stimulation with TNF-α, indicating that this signalling pathway is functional in both organoids. Thus our results suggest that the observed differences in the reporter organoid responsiveness would be associated with another factor. In this regard, Carlsen and colleagues reported that the small intestine of NF-κB -RE-Luc mice showed the largest luminescent signal by in vivo imaging after LPS challenge. However, ex vivo luciferase signal was lower at the colon compared with the small intestine^21^. These data match our findings, suggesting that the difference in reporter sensitivity along the intestine may be an intrinsic characteristic of the animal model rather than the organoid culture. Moreover, the similarities in the reporter activity between the in vivo and our ex vivo models support the ability of the intestinal organoids to closely recreate tissue behavior, reinforcing the value of this tool as an alternative to the use of laboratory animals.

Despite the ability of TNF-α to elicit a clear NF-κB reporter response in jejunum-derived organoids, other proinflammatory stimuli, usually present in the intestinal environment like LPS, IL-1β or inactivated *S. enterica* failed to induce a detectable response.

It is known that Toll-like receptors (TLR) and the IL-1 family Receptors (IL-1R) have remarkable structural similarity and their activation culminates in the expression of genes encoding pro-inflammatory cytokines and chemokines through activation of NF-κB^26^. Nevertheless, other authors have also reported a lack of response of small intestinal organoids to LPS and IL-1β, suggesting that it could be attributed to the low expression levels of TLR and IL-1R in the epithelium^27^. It is possible that a similar explanation or a lower accessibility of the compounds to the surface receptors could have affected the response. In the present approach, the apical surface of the epithelium is inside the organoids structures, while the basolateral side is exposed to the matrix, in more direct contact with the stimuli added to the media. Future studies reducing the incubation time or applying other methodological approximations to access the luminal area (i.e. microinjection, two dimensional cultures) would contribute to clarifying this issue.

Inflammation plays a key role in the development of intestinal diseases such as inflammatory bowel disease and gastrointestinal malignancy. The development of in vitro models that closely recreate the physiology and cellular composition of the tissue allows test results with higher predictive value than 2D models. Herein, in order to validate the usefulness of our model, TNF-α-induced reporter organoids were challenged with a variety of anti-inflammatory compounds. All of them elicited a significant decrease in the luciferase activity, demonstrating the ability of the model to evaluate putative NF-κB inhibitors. Dexamethasone is a potent glucocorticoid known to inhibit NF-κB activation by increasing the nuclear export rate of activated p65 NF-κB subunit^28^. On other hand, Bay11-7082 has multiple targets, among which the inhibition of IκB kinase and the consequent inhibition of the translocation of p65 NF-κB to the nucleus has been well documented^29^. Regarding natural compounds, our results suggest that both probiotics strains would secrete factors capable of modulating NF-κB activation in the intestinal epithelial cells. It has been described that cell-free supernatants from *L. plantarum* can inhibit NF-κB pathways in intestinal cells by preserving its inhibitor IkappaBalpha protein from degradation and inhibiting the activity of the proteasome^30^. Besides the multiple reported beneficial effects of *L. reuteri* colonization in intestinal inflammatory diseases^31^, it has also been shown that some strains can produce soluble metabolites with anti-inflammatory properties. For instance, the CRL 1098 strain has shown to produce factors that inhibit NF-κB pathway in macrophages, reducing LPS- induced inflammation in lungs^32^, whereas the 6475 strain produces histamine, which inhibits TNF-α production in THP1 cells^33^.

Concerning Vioprolide A, even though the molecular mechanisms of its biological effects are not completely elucidated, our results add new data reporting an inhibitory effect of this compound over the NF-κB signaling in intestinal epithelial cells which are in line with our previous reported results on 2D cultures^14^.

Taken together, these results demonstrate that these reporter intestinal organoids could be a useful tool for detecting anti-inflammatory agents acting at different steps in the NF-κB pathway in the small intestine.

Equally important, this model represents an alternative method contributing with the ¨3Rs¨ concept of Russel and Burch in animal experimentation^34^. Characteristics such as natural self-renewal capability, unlimited proliferation and a similar composition and architecture to the primary tissue, make intestinal organoids a suitable strategy for the partial replacement and the reduction in the use of laboratory animals.

## Conclusion

The NF-κB-RE-Luc-reporter organoids provide a novel strategy for exploring the activation or inhibition of TNF-α-induced NF-κB signaling at the small intestinal epithelium while contributing to reducing the use of laboratory animals. Implementation of these models in co-culture systems with immune cells and/or microorganisms and the use of gene editing tools to alter specific steps in the NF-κB pathway are examples of future applications to deepen the understanding of the role that this transcription factor plays in the physiology and pathology of the small intestinal tissue.

## Methods

### Reagents

All chemicals used were of the highest available grade and purchased from Sigma Aldrich, Merck (USA). Culture media, fetal bovine serum, and consumables for cell culture were obtained from Gibco, ThermoFisher Scientific (USA); plasticware was obtained from Corning (USA). Basement membrane extract Cultrex Type II was obtained from R&D Systems (USA); gastrin I, SB 431542 (TGF-β inhibitor), and Y-27632 (ROCK inhibitor) were purchased from Peprotech (USA). IL-1β was from Invitrogen (USA); LPS and TNF-α were obtained from Sigma.

### Ethics statement and animals management

The experimental protocols were approved by the institutional *Comisión de Ética en el Uso de Animales* (CEUA) (protocol #002-21) and were performed according to national law #18.611 and relevant international laboratory animal welfare guidelines and regulations. In addition, the ARRIVE guidelines were followed to carry out the present study. NFκB-RE-luc transgenic mice (model # 10499, Taconic, USA) and wild type BALB/cJ were bred at the Laboratory Animals Biotechnology Unit of the Institut Pasteur de Montevideo under specific pathogen-free conditions in individually ventilated racks (IVC, 1285L, Tecniplast, Milan, Italy). Animals were euthanized with CO2 and intestine biopsies were removed to establish organoid cultures.

### Intestinal organoid culture

Intestinal crypts were isolated from duodenum, jejunum, ileum, and colon regions of 6–8 weeks-old male and female BALB/cJ and NFKB-RE-luc transgenic mice. This transgenic model was generated by Carlsen et al.^21^ and has a randomly integrated DNA construct containing 6 NFκB-responsive elements (RE) from the CMVα (immediate early) promoter placed upstream of a basal SV40 promoter, and a modified firefly luciferase cDNA (Promega pGL3). Tissue biopsies of 3 cm length were obtained from each intestinal region and flushed with ice-cold sterile phosphate buffered saline (PBS) supplemented with 1% w/v penicillin/streptomycin to remove luminal contents. The intestine was then opened longitudinally and cut into 0.5 cm pieces followed by washing in cold PBS until the supernatant was clear. Tissue fragments were then incubated with 10 mM ethylene diamine tetra acetic acid (EDTA) for 20 min with gentle shaking.

After removing the EDTA, the fragments were resuspended in PBS supplemented with 0.1% bovine serum albumin (BSA) and pipetted up and down with a 10 mL pipette five times to ensure crypt release. Crypt-containing fractions were pooled together, passed through a 70 μm cell strainer, and centrifuged at 200 × *g* for 5 min at 4 °C. The crypt pellet was resuspended in extracellular matrix Cultrex (250 crypts/20 μL matrix). The crypt/matrix suspension was plated in a 6 multi-well plate (6 drops of 20uL per well) and incubated for 5–10 min at 37 °C for the matrix to polymerize. Subsequently, 2 mL of organoid medium was added per well (Advanced DMEM/F12, 1% w/v l-glutamine, 1% w/v penicillin/streptomycin, 50% L-WRN conditioned media, and 10 μM Y-27632). The L-WRN media was produced in our laboratory by the commercially available cell line L-WRN (ATCC 3276) as indicated by the provider. The conditioned media produced by this cell line constitute a source for recombinant Wnt-3A, R-spondin 3, and Noggin proteins. The medium was also supplemented with 10 μM SB 431,542 (for small intestinal organoids) or 10 nM gastrin (for colon-derived organoids). Organoids were incubated at 37 °C in a 5% CO_2_ humidified atmosphere. For maintenance, the medium was renewed every 3 days with organoid media without inhibitors. Organoids were split every 4–7 days in a ratio of 1:2. All the assays were performed using organoids in passages 4–12, at day 4 of culture, unless otherwise specified. Organoids were imaged on days 1 and 4 of culture using a bright field of an inverted microscope Olympus IX81 (Japan) equipped with 10× objective lenses. Images were acquired with µManager software and analyzed with ImageJ software^35^.

### Colony forming assay, morphological parameters and histological analysis

Intestinal organoids obtained from two mice were dissociated into single cells using TrypLE Express and further filtered using a 50-µm cell strainer. One thousand single cells were plated in 7 µL of matrix per well in a 96 multiwell plate and cultured in organoid media as described above. Images of six wells per each intestine region-derived organoid were captured on day 4 and 8 post-seeding with 10× objective lenses in a bright-field microscope. Replication capacity was evaluated as the number of spheroids generated from 1000 seeded single epithelial cells, quantified on day 4 of culture. Circularity and area of the organoids were measured on days 4 and 8 of culture. On day 8 of culture, the number of buds per organoid was manually counted. All image analyses were performed with Fiji^36^.

For histological analysis, organoids derived from jejunum and colon were fixed at day 4 of culture in PFA4% for 20 min at 4 °C, washed twice with PBS and stained with Methylene Blue solution to facilitate visualization. Samples were washed and resuspended in ethanol 70%, dehydrated and embedded in paraffin. Hematoxylin and eosin-stained (HE) sections were prepared by a routine method and read under a light microscope.

### RNA isolation and qPCR analysis

Total RNA from intestinal tissue and organoids at day 4 of culture was purified using the Monarch total RNA extraction kit, according to the manufacturer's protocol (New England Biolabs, USA). Concentration of RNA in samples was determined using a spectrophotometer DS-11Fx (DeNovix, USA). For reverse transcription, 0.5 μg of total RNA was transcribed using the M-MLV Reverse Transcriptase, according to the protocol (Bio-Rad: Cat.# 1708841). Quantitative RT–PCR was performed on a thermocycler QuantStudioTM 3 (Thermofisher Scientific, USA), using FastStart Universal SYBR Green master (Roche), 200 nM of each specific primer (excepting primers for Chromogranin A, which were used at 60 nM), and 1μL of cDNA. The following cycling parameters were used for all the markers: 1 cycle of 95 °C for 4 min; 40 cycles of 95 °C for 10 s, 60 °C for 20 s and 72 °C for 30 s; 1 cycle of 72 °C for 5 min; melting curve analysis over a temperature range of 95 to 60 °C. Primers were synthesized by IDT (sequences are listed in Supplementary Table S2). Actin was used as a reference gene. Relative gene expression levels were determined using the standard curve method. Standard-curve points and unknown samples were performed in technical triplicates and replicates, respectively, and at least two biological replicates were analyzed per experimental condition.

### Luciferase reporter assay

Organoids were split 1:2 and seeded in Cultrex (7 μL/well) in 96 well plates in 0.15 mL of organoid medium. At day 4 of culture, medium was replaced for assay media (Advanced DMEM/F12 containing 1% w/v l-glutamine, 1% w/v penicillin/streptomycin, 10 mM HEPES and 1% v/v N2 supplement and 1% v/v B27 serum-free supplement) and organoids were stimulated with different concentrations of pro-inflammatory stimuli: 1–100 ng/mL TNFα, 1–10 μg/mL LPS, 1/2–1/500 dilutions of heat inactivated *Salmonella enterica* (10^8^ CFU equivalent), 1–100 ng/mL IL-1β. Each condition was assayed in triplicate. After 24 h of incubation at 37 °C in a 5% CO2 humidified atmosphere, the medium was removed, 100 μL of 1 mM luciferin solution (PerkinElmer, USA) was added to each well, and luciferase activity was measured in a luminometer (BMG Labtech, UK). The arbitrary light units from each well were normalized by the absorbance at 570 nm obtained from an 3-(4,5-dimethylthiazol-2-yl)-2,5-diphenyl-2H-tetrazolium bromide **(**MTT) assay for each corresponding well. Unstimulated organoids were used as negative controls. Activation of NF-κB transcription factor was expressed as fold change of the normalized signal with respect to the unstimulated group.

Validation assays of the reporter organoids were performed by evaluating the anti-inflammatory activity of known NF-κB inhibitors. Dex 10 μM and Bay 10 μM were used as synthetic inhibitors; VioA, as a natural peptide already tested in NF-κB reporter human intestinal cell lines^14^; and conditioned media obtained from *Lactobacillus reuteri* and *Lactobacillus plantarum*. Both Lactobacillus strains are present in the intestine of healthy humans and their anti-inflammatory action has been well documented^37,38^. Organoids were stimulated with 10 ng/mL TNF-α and the indicated anti-inflammatory compounds. After 24 h of incubation, luciferase activity was measured as described previously. Each condition was assayed in triplicate. Cells without treatment and cells treated only with TNF-α or the different compounds were included as controls.

The stability of the reporter response was verified using jejunum-derived organoids obtained from the same animal, at different passage numbers. Organoids were thawed at passage 7 and luciferase reporter stability was compared with cultures that had undergone a weekly splitting (passage 9, 12, 13 and 16). Data was normalized and the half maximal effective concentration (EC50) was determined using nonlinear regression 3-parameter fit.

### Lactobacilli conditioned media preparation

*Lactobacillus reuteri (*ATCC 23272) and *Lactobacillus plantarum* (ATCC 8014) were incubated at 37 °C for 24 h in de Man, Rogosa and Sharpe agar (MRS) broth, then diluted in culture broth according to ATCC guidelines. For Lactobacilli conditioned media preparation, 3 mL of a log phase culture of *L. reuteri*or *L. plantarum* (optical density at λ600 nm about 0.6) were inoculated in 50 mL Advanced DMEM/F12 culture media and incubated at 37 °C for 6 h. The media was centrifuged twice, adjusted to pH 7.4, then filtered through a 0.22 μm filter to remove live bacteria and stored at − 20 °C until used in validation assays.

### NF-κB p65 subunit translocation assay

Organoids obtained from colon and jejunum regions were seeded in a 6 multi-well plate (6 drops of 20 μL Cultrex/well) in organoid medium. After 4 days of culture, the medium was renewed with an assay medium supplemented with TNF-α (50 ng/mL) and further incubated for 3 h. Then, organoids were fixed and processed for the immunofluorescence detection of p65. Quantification of the NF-κB nuclear translocation was performed using the Intensity Ratio Nuclei Cytoplasm Tool (Image J). The ratio between the cytoplasm and the nuclei intensity signal (N/C ratio) was defined as the percentage of nuclei over the percentage of cytoplasm intensity signals.

### Immunofluorescence assays

For the immune detection of p65 , Lysozyme and Ki67, organoids were removed from the matrix and fixed with 4% v/v Paraformaldehyde (PFA) solution at room temperature (RT) for 30 min. Permeabilization was performed at RT for 15 min with 0.5% v/v Triton X-100 in PBS and then blocked with 2% w/v BSA in PBS for 1 h at RT. Samples were incubated with the primary antibody (Anti-NF-κB-p65 ab7970 or anti-Ki67 ab 15580, from Abcam or anti-Lysozyme A0099, from Dako) and methyl green (1:5000 dilution) in 2% BSA, 0.1% v/v Triton X-100 in PBS overnight at 4 °C. After incubation, organoids were washed and incubated with secondary antibody anti-rabbit Alexa Fluor 488 (Invitrogen, USA) (1:1000 dilution) and Phalloidin Texas Red for 1 h in the dark at RT. Cells were mounted on a cover slide using ProLong^®^ Gold Antifade Reagent (ThermoFisher Scientific, USA). All images were obtained using laser confocal microscope Zeiss LSM 880 equipped with 25X and 40X objectives and 488 nm and 631 nm lasers. Images were processed using ImageJ software.

### Statistical analysis

NF-κB activation data was expressed as the mean ± SD values of triplicates from one representative experiment, out of three independent experiments executed, unless otherwise indicated. The One-Way ANOVA test with Tukey's post hoc test were used to evaluate multi-comparison differences. Quantitative PCR data and immunofluorescence scores were analyzed using a 2-tailed t-test. GraphPad Prism Software version 6.00 was used for statistical calculations. In all cases, differences were considered statistically significant when *P* < 0.05.

## Supplementary Information

Supplementary Information.
