# Supplementary material for: Jejunum-derived NF-κB reporter organoids as 3D models for the study of TNF-alpha-induced inflammation
Source: Sci Rep. 2022 Aug 24;12:14425. doi: 10.1038/s41598-022-18556-3 (PMC9829906; doi:10.1038/s41598-022-18556-3)
Supplement: Supplementary file 1 — Supplementary Information. [file 41598_2022_18556_MOESM1_ESM.pdf]

Jejunum-derived NF-κB reporter organoids as 3D models for the study of TNF-alpha-induced inflammation.

Hellen Daghero, Flora Doffe, Belén Varela, Victoria Yozzi, José Manuel Verdes, Martina Crispo, Mariela Bollati-Fogolín and Romina Pagotto

Supplementary Materials

Comparison of TNF-α-induced reporter response from different intestinal region-derived organoids.

Organoids derived from duodenum, jejunum, ileum and colon were obtained from the transgenic reporter mice NF-kB hRE-Luc. They were stimulated with different concentrations of TNF-α and 24 h later, luciferase activity was measured as the mean of NF-kB activation. When possible, data presented in **Figure 4b** from the manuscript were fit to a 3 parameters dose-response curve using GraphPad Prism software. Results showed that jejunum-derived organoids best fit the model, with an R square of 0.916 and an EC50 of 3.66 ng/mL (Table S1).

Regarding the reporter response elicited by TNF-α 10ng/mL, jejunum and ileum derived-organoids response was statistically different from colonoids (ANOVA and Tukey's multiple comparison-test; $^*P < 0.05$), which were unresponsive to the stimulus (Figure S1).

Parameter	Organoids derived from			
	Duodenum	Jejunum	Ileum	Colon
EC50	12.11	3.66	0.12	-
CI 95%	1.64-8.16	0.98-14.9	very wide	-
R2	0.54	0.92	0.47	-

Table S1: TNF-α concentration-response curve fitting parameters from different intestinal region-derived-organoids. The half-maximal effective concentration (EC50) to TNF-α, the corresponding 95% confidence interval (CI 95%) and the R square of the curve is indicated for each type of organoid. Data was obtained from triplicates from one representative experiment.

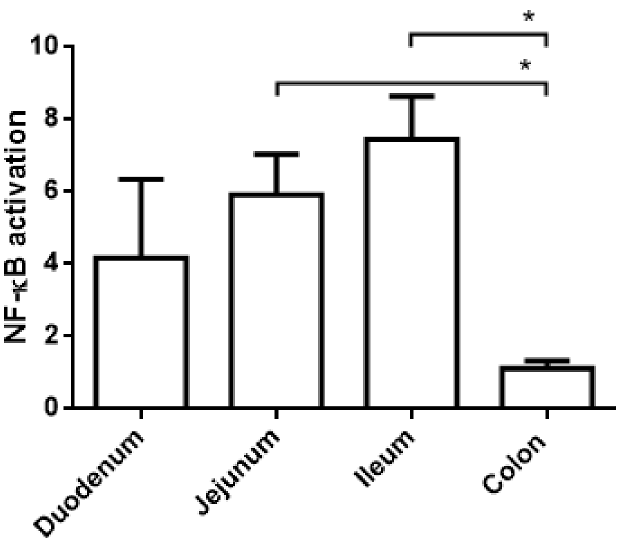

Figure S1: Comparison of the reporter response of different intestinal region-derived-organoids to TNF-α 10 ng/mL. Data was expressed as mean ± SD of triplicates from one representative experiment. *P< 0.05, significant differences in the NF-kB activation level attained between the indicated type of organoids.

Comparison of TNF-α-induced reporter response from the same intestinal region-derived organoids.

Duodenum-, jejunum-, ileum- and colon-derived organoids obtained from different NF-kB hRE-Luc mice (n=3 for each region) were stimulated with TNF-α 10 ng/mL and 24 h later, luciferase activity was measured. Results showed that jejunum and ileum responses to TNF-α were significantly higher compared to the unstimulated organoids (ANOVA and Tukey's multiple comparison-test; #P< 0.05). Nevertheless, the maximum level of NF-kB activation attained was different between animals (ANOVA and Tukey's multiple comparison-test; *P< 0.05). Two organoids did not differ from the control value regarding duodenum, whereas colon-derived organoids were unresponsive in all cases (Figure S2).

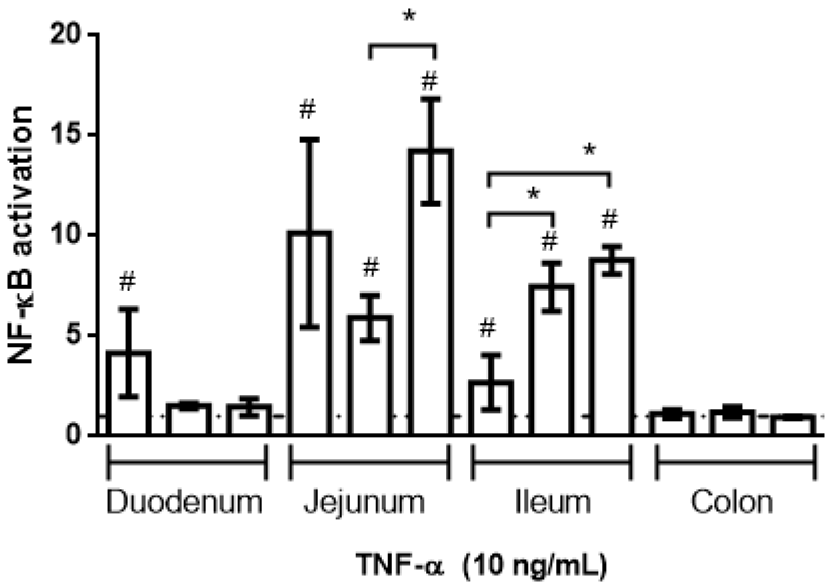

Figure S2: Comparison of the reporter response of organoids from different animals to TNF-α 10 ng/mL. Organoids derived from different NF-kB hRE-Luc mice were stimulated with TNF-α 10 ng/mL and the reporter response was measured. NF-κB activation was expressed as fold change with respect to the unstimulated control. Data was expressed as mean ± SD of triplicates. *P< 0.05, significant differences between indicated bars; #P< 0.05, significant differences with respect to the unstimulated control from the same intestinal region-derived organoid.

Gene (mouse)	Forward Primer	Reverse Primer	NM number
**Chromogranin A**	5´AGACTACAGACCCACTCCCG 3´	5´AGATGACTTCCAGGACGCAC 3´	NM_007693.2
**Lgr5**	5´TCTCCTACATCGCCTCTGCT 3´	5´TTCCTCCGGAACCTGTCTCA 3´	NM_010195
**Lysozyme**	5´AACTACAACCGTGGAGACCG 3´	5´TGCAATTGATCCCACAGGCA 3´	NM_0.13590.4
**Mucin 2**	5´AAACTGCTCTCTGGACTGCC 3´	5´TTGGTTGGTGTGCTGAGTGT 3´	NM_023566.3
**Villin**	5´ATTCCTTCAGACGCATGCCA 3´	5´GCCCTCCCTTTTGAGTGTGA 3´	NM_009509.2
**β-actin**	5´GCAGGAGTACGATGAGTCCG 3´	5´ACGCAGCTCAGTAACAGTCC 3´	NM_007393.5
**TNF-R1/ Tnfrsf1a**	5´GATCCCCTGCCTGTCAAAGA 3´	5´TCGCAAGGTCTGCATTGTCA 3´	NM_011609.4
**TNF-R2/ Tnfrsf1b**	5´GATGCCAAGGTGCCTCATGT 3´	5´TGGCTTCCGTGGGAAGAATC 3´	NM_011610.3

Table S2: Primers used for real-time RT-PCR experiments
